# High-precision intraoperative diagnosis of gliomas: integrating imaging and intraoperative flow cytometry with machine learning

**DOI:** 10.3389/fneur.2025.1647009

**Published:** 2025-09-09

**Authors:** Shunichi Koriyama, Yutaka Matsui, Takahiro Shioyama, Mikoto Onodera, Manabu Tamura, Tatsuya Kobayashi, Buntou Ro, Kenta Masui, Takashi Komori, Yoshihiro Muragaki, Takakazu Kawamata

**Affiliations:** ^1^Department of Neurosurgery, Tokyo Women’s Medical University, Tokyo, Japan; ^2^Technical Department, Atom Medical Corporation, Tokyo, Japan; ^3^Ogino Memorial Laboratory, Nihon Kohden Corporation, Tokyo, Japan; ^4^Faculty of Advanced Techno-Surgery (FATS), Graduate School of Medicine, Institute of Advanced Biomedical Engineering and Science, Tokyo Women’s Medical University, Tokyo, Japan; ^5^Department of Integrated Neuroscience, Graduate School of Biomedical Sciences, Nagasaki University, Nagasaki, Japan; ^6^Department of Pathology, Tokyo Women’s Medical University, Tokyo, Japan; ^7^Department of Laboratory Medicine and Pathology, Tokyo Metropolitan Neurological Hospital, Tokyo Metropolitan Hospital Organization, Tokyo, Japan; ^8^Department of Medical Device Engineering, Graduate School of Medicine, Kobe University, Kobe, Japan

**Keywords:** glioma, machine learning, magnetic resonance imaging, methionine positron emission tomography, intraoperative flow cytometry

## Abstract

**Introduction:**

Accurate intraoperative identification of glioma molecular subtypes, such as isocitrate dehydrogenase mutation and 1p/19q co-deletion, is essential for precise diagnosis, prognostication, and determining the extent of tumor resection—balancing maximal tumor removal with preservation of neurological function.

**Methods:**

We developed a machine learning model that integrates preoperative imaging features [magnetic resonance imaging, computed tomography, and ^11^C-methionine positron emission tomography (PET)] and intraoperative flow cytometry (iFC) data to predict molecular subtypes of glioma in real-time.

**Results:**

Analyzing 288 cases of diffuse gliomas, this model achieved an overall accuracy of 76.0%, with a macro-average ROC-AUC of 0.88 and a micro-average ROC-AUC of 0.89. Key predictive factors included the tumor-to-normal uptake ratio on PET, malignancy index from iFC, and patient age, all of which showed significant differences between correctly and incorrectly classified cases. We also developed a prototype application that visualizes the prediction results intraoperatively, thereby supporting real-time surgical decision-making.

**Conclusion:**

This integrated approach enhances the precision of intraoperative molecular diagnosis and has the potential to optimize surgical strategies for glioma treatment.

## 1 Introduction

The 2021 World Health Organization (WHO) Classification of Tumors of the Central Nervous System (CNS) defines gliomas by their molecular genetic alterations, such as IDH1/2 mutations and 1p/19q co-deletion (1, 2). These molecular subtypes are essential for diagnosis and closely associated with prognosis and recurrence risk. They also influence surgical strategies; some subtypes benefit from aggressive resection, whereas others require more conservative approaches to preserve neurological function. Therefore, determining the molecular subtype preoperatively or intraoperatively is critical.

Molecular classification is also closely associated with the extent of resection (EOR) and overall survival. In glioblastoma (GBM), gross total resection is correlated with prolonged survival, especially in isocitrate dehydrogenase (IDH)-wild-type GBM, as shown in recent systematic reviews (3–5). For low-grade gliomas (LGGs), particularly astrocytomas, a resection rate exceeding 90% significantly improves long-term outcomes (6–8). Conversely, no clear survival benefit has been observed with EOR in oligodendrogliomas, likely because of their increased chemosensitivity (9). In tumors located near eloquent regions such as the language or motor cortex, functional preservation is prioritized, and techniques such as intraoperative mapping and monitoring are essential to achieve maximal safe resection (10).

In response to these challenges, machine-learning-based approaches that predict molecular subtypes noninvasively from magnetic resonance imaging (MRI) and positron emission tomography (PET) derived radiomic features have gained attention. Our group previously developed a deep learning model using T1-, T2-, and fluid attenuated inversion recovery (FLAIR) weighted MRI to predict the molecular subtypes of LGGs, achieving an accuracy of 68.7% (11). An area under the curve (AUC) of 0.89 has also been reported for predicting IDH mutations using radiomics from ^11^C-methionine PET (^11^C-MET PET) (12). A recent systematic review provides a comprehensive overview of machine- and deep-learning approaches for glioma molecular subtyping (13). In addition to imaging-based approaches (14), we also explored the use of intraoperative flow cytometry (iFC) as a rapid diagnostic tool that enables the quantification of DNA content, aneuploidy, and S-phase fraction within minutes. iFC has been shown to assist in malignancy assessment, resection margin determination using the malignancy index (MI), and prognostication based on DNA ploidy (15–17). We also demonstrated that iFC allows intraoperative differentiation between GBM and primary central nervous system lymphoma and proposed surgical strategies for LGGs that integrate molecular data obtained from iFC (18, 19).

In this study, we developed a novel machine learning-based prediction model that integrates preoperative imaging features (MRI, CT, and ^11^C-MET PET) with iFC-derived histograms and ploidy data to enable real-time prediction of molecular subtypes, specifically IDH mutations and 1p/19q co-deletions. This approach moves beyond conventional iFC-based malignancy grading and provides an innovative framework for intraoperative molecular classification by combining cellular and radiological data.

Furthermore, we implemented this model in a prototype application designed for intraoperative use and evaluated its clinical feasibility. Our approach may contribute to improving the accuracy of intraoperative molecular diagnosis and support surgical decision-making in glioma treatment.

## 2 Methods

### 2.1 Patient cohort

This retrospective study included 288 patients with newly diagnosed diffuse gliomas who underwent surgical resection at Tokyo Women’s Medical University Hospital between 2016 and 2024. All tumors were classified according to the WHO 2021 CNS tumor classification system.

The IDH1 mutation status was initially evaluated using immunohistochemistry (IHC), and cases negative for IDH1-R132H were further analyzed for both IDH1 and IDH2 mutations using direct sequencing. 1p/19q co-deletion status was assessed using either fluorescence *in situ* hybridization or multiplex ligation-dependent probe amplification. Based on integrated histological and molecular diagnoses, the dataset included 141 IDH-wild-type astrocytomas (Astro-WD), 68 IDH-mutant astrocytomas (Astro-MT), and 79 oligodendrogliomas (Oligo). All patients underwent iFC analysis of tumor samples obtained during surgery.

### 2.2 Feature extraction and preprocessing

Preoperative neuroimaging features were extracted from MRI, CT, and ^11^C-Met-PET scans, including the presence or absence of a T2-FLAIR mismatch, gadolinium enhancement, calcification on either T2*-weighted MRI or CT, and tumor-to-normal uptake ratio (TNR) on MET PET. Patient age was also included as a clinical variable. Each imaging feature was independently evaluated by three board-certified neurosurgeons and a majority vote was used to finalize the label.

T2-FLAIR mismatch, enhancement, and calcification were assessed using a standardized three-point scale: “+” (clearly present), “+/−” (ambiguous or mild), and “−” (clearly absent). Intraoperative tumor samples were processed using iFC to generate nuclear DNA histograms (binned into 10 intervals from the original 512-bin signal), detect aneuploidy, and compute the MI. Continuous variables (age, TNR, and MI) were normalized using *Z*-score standardization, and categorical features (four imaging variables and aneuploidy) were one-hot encoded. All features were then concatenated into a single fused vector as input to the machine-learning models.

### 2.3 Model development and evaluation

The classification task aimed to predict molecular subtypes across three classes: Astro-WD, Astro-MT, and Oligo. We employed a Random Forest as the benchmark model, with key hyperparameters such as the number of estimators and the splitting criterion optimized within each cross-validation fold. Model performance was evaluated using stratified five-fold cross-validation with a fixed random seed for reproducibility (20). As a deep learning comparator, we implemented TabNet, a state-of-the-art interpretable model optimized for structured and tabular data (21). TabNet leverages sparse attention mechanisms to dynamically select relevant features at each decision step.

**Figure 1:**
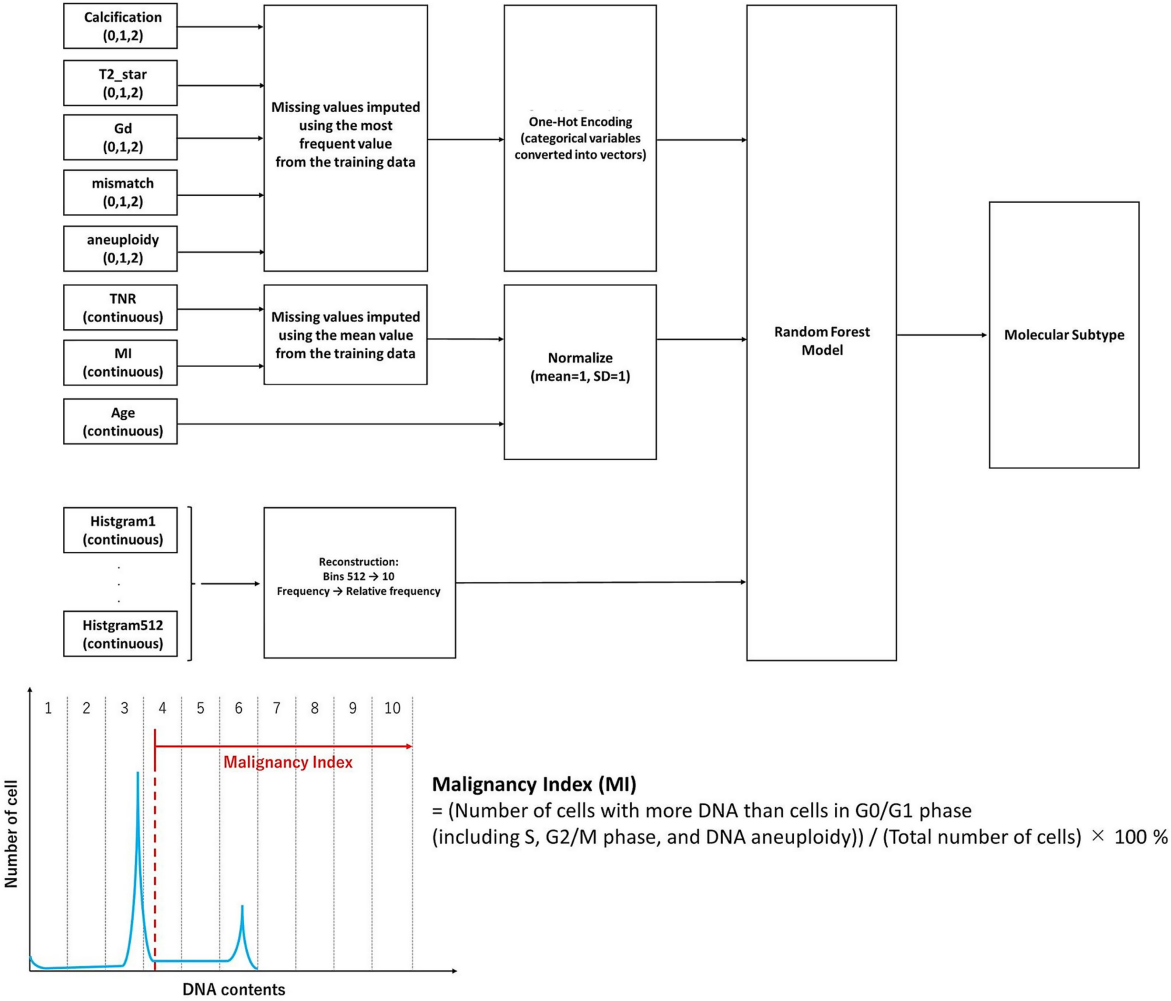
Overview of the machine learning pipeline for intraoperative molecular classification of gliomas. Categorical features (calcification, T2*star, gadolinium enhancement, fluid attenuated inversion recovery mismatch, and aneuploidy) were one-hot encoded, and continuous features (tumor-to-normal uptake ratio, malignancy index, and age) were normalized. The histogram data (originally 512 bins), obtained from intraoperative flow cytometry representing the DNA content distribution of tumor cells, were downsampled to 10 aggregated features based on relative frequencies. The malignancy index was defined as the proportion of cells with more DNA content than those in the G0/G1 phase, including cells in S phase, G2/M phase, and those with DNA aneuploidy. All features were integrated and input into a Random Forest model to classify glioma molecular subtypes. The missing values were imputed using the mean of the training set. The model performance was evaluated using five-fold stratified cross-validation. The synthetic minority oversampling technique was applied to the training data to address class imbalance.

Training was conducted using a batch size of 128 and 10 decision steps, and an AdamW optimizer with early stopping (patience = 10 epochs) to avoid overfitting. The attention-based feature selection process in TabNet also enables the visual interpretability of predictive signals (22). Classification performance was evaluated using accuracy, *F*_1_-score, and receiver operating characteristic (ROC)-AUC. Statistical comparison between models was performed using the DeLong test. Feature importance was assessed using the permutation importance for Random Forest and attention weights for TabNet (23). An overview of the data processing pipeline, including imaging and iFC-derived features and model architectures, is shown in Figure 1.

### 2.4 Ethical considerations

This study was approved by the Institutional Review Board of Tokyo Women’s Medical University (Approval No. 3540-R6). Written informed consent was obtained from all participants in accordance with the institutional and national guidelines.

## 3 Results

### 3.1 Classification accuracy for molecular subtypes

We developed classification models for three molecular subtypes—Astro-WD, Astro-MT, and Oligo—using integrated features derived from preoperative imaging (gadolinium enhancement, T2-FLAIR mismatch, calcification on T2*-weighted MRI or CT, and TNR on ^11^C-MET PET), iFC (DNA histogram, aneuploidy status, MI), and patient age.

To ensure the reliability of radiological annotations, inter-rater agreement among the three neurosurgeons was assessed using weighted Cohen’s kappa statistics. Substantial agreement was observed for gadolinium enhancement (*κ* = 0.859) and calcification (*κ* = 0.717), while T2 hypointensity (*κ* = 0.485) and the T2-FLAIR mismatch sign (*κ* = 0.452) showed moderate agreement. These results confirm that the imaging features used for model input were reproducible and clinically reliable.

**Figure 2:**
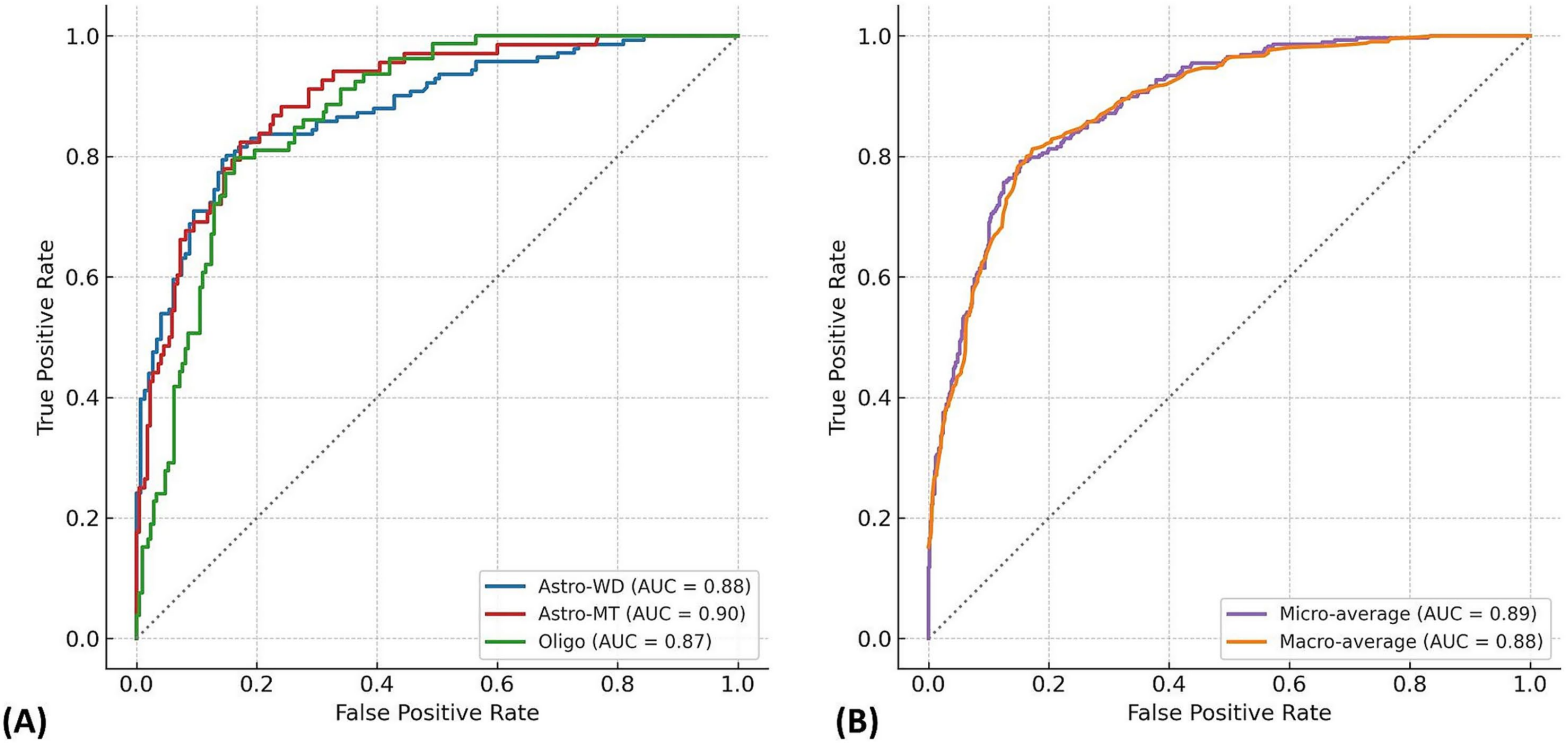
Multi-class ROC curves for molecular subtype prediction using the Random Forest model. **(A)** Receiver operating characteristic (ROC) curves are shown for each glioma molecular subtype: IDH-wild-type astrocytoma (Astro-WD), IDH-mutant astrocytoma (Astro-MT), and oligodendroglioma (Oligo), based on the Random Forest classifier trained on 288 cases. The AUC values were 0.88 for Astro-WD (blue), 0.90 for Astro-MT (red), and 0.87 for Oligo (green), indicating robust discriminatory performance across all subtypes. **(B)** Micro-average and macro-average ROC curves summarize overall classification performance. The micro-average AUC (0.89) reflects performance across all individual predictions, whereas the macro-average AUC (0.88) represents the unweighted mean of AUCs for each class. The near-overlapping curves demonstrate stable and balanced prediction capability of the model.

In the dataset of 288 cases, the Random Forest model achieved an overall classification accuracy of 76.0%, with a macro-average ROC-AUC of 0.88 and micro-average ROC-AUC of 0.89 (Figure 2). The *F*_1_-scores were 0.82 for Astro-WD, 0.72 for Astro-MT, and 0.68 for Oligo, indicating favorable classification performance across all three subtypes. The sensitivity and specificity were also stable, as shown in Table 1 and the confusion matrix in Table 2.

**Table 1:** Performance metrics of the Random Forest model for molecular subtype classification.

Subtype	Sensitivity	Specificity	*F*_1_-score
A-WD	0.81	0.84	0.82
A-MT	0.72	0.91	0.72
Oligo	0.70	0.87	0.68

The table shows the classification performance for each molecular subtype, including sensitivity, specificity, precision, *F*_1_-score, and support (number of true cases), based on stratified five-fold cross-validation. The overall accuracy of the model was 0.76, indicating consistent and balanced prediction performance across all three classes. A-WD, IDH-wild-type astrocytoma; A-MT, IDH-mutant astrocytoma; and Oligo, oligodendroglioma.

**Table 2:** Confusion matrix of Random Forest predictions for molecular subtypes.

Imaging+Age+iFC model		Predict		
		A-WD	A-MT	Oligo
Molecular subtype	A-WD	114	9	18
	A-MT	10	49	9
	Oligo	14	10	55

The table displays a confusion matrix comparing the predicted labels with the true molecular subtype labels. Diagonal values represent correct classifications, whereas off-diagonal values indicate incorrect classifications. The confusion matrix provides a comprehensive view of the strengths of the model and areas that require improvement. A-WD, IDH-wild-type astrocytoma; A-MT, IDH-mutant astrocytoma; and Oligo, oligodendroglioma.

The macro-average ROC-AUC represents the unweighted mean of the AUC values across all classes, offering a balanced measure of model performance. By contrast, the micro-average ROC-AUC was calculated from pooled predictions and reflected class imbalance, making it more representative of real-world performance. These values are summarized in Figure 2.

To evaluate the contribution of each modality, we trained models using limited feature sets. The imaging + age model achieved 66% accuracy, while the iFC + age model reached 72%. The full model combining imaging, iFC, and age achieved 76%. Although the imaging + age model (66%) underperformed compared to imaging alone (69%), this outcome should not be interpreted as evidence that adding age universally degrades imaging-based prediction performance. It likely reflects interactions specific to the current model or dataset configuration. Notably, the addition of iFC data improved the overall classification accuracy by approximately 7 percentage points compared to imaging features alone. These findings highlight the complementary roles of imaging and iFC features, as well as the additive value of age. Confusion matrices are provided in Supplementary Table 1.

### 3.2 Feature importance and contribution of flow cytometry

**Figure 3:**
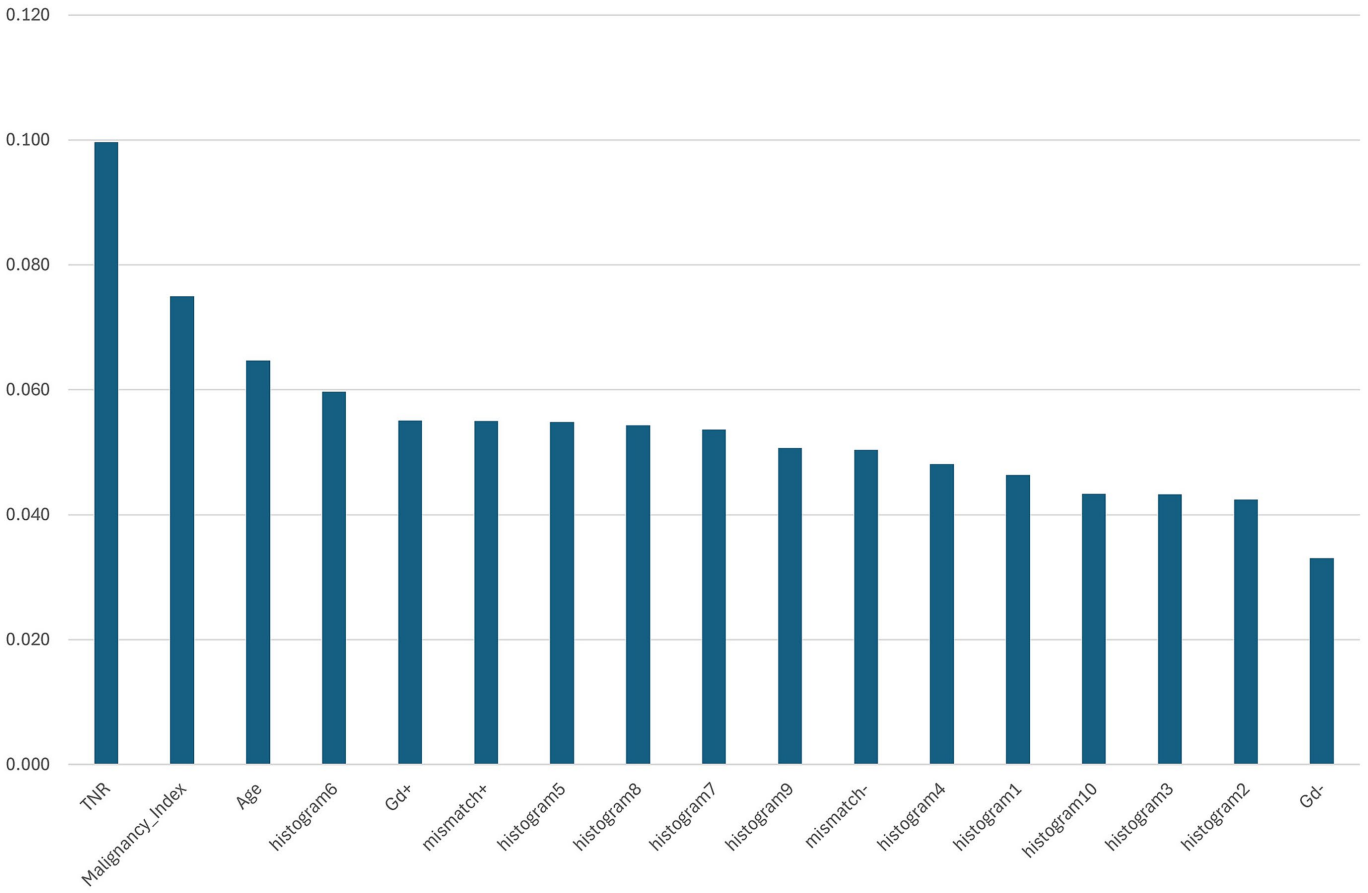
Feature importance ranking based on mean Gini importance across five-fold cross-validation. The bar chart shows the relative importance of each input feature used in the Random Forest model to predict glioma molecular subtypes. The tumor-to-normal uptake ratio (TNR) from ^11^C-methionine positron emission tomography exhibited the highest importance, followed by the malignancy index (MI), patient age, and histogram bin 6 derived from intraoperative flow cytometry (iFC). Other significant features included gadolinium enhancement (Gd+), T2-FLAIR mismatch (mismatch+), and additional histogram bins. These results highlight the combined contribution of imaging and iFC-derived features for molecular subtype classification.

The feature importance was evaluated using the Gini importance (mean decrease in impurities) calculated during Random Forest training (Figure 3). The most influential feature was the TNR on MET-PET, followed by the MI, age, DNA histogram bin 6, and gadolinium enhancement. These features are thought to reflect the key biological properties associated with tumor metabolism, cellular proliferation, patient characteristics, and vascular permeability.

In particular, bin 6 of the DNA histogram made a substantial contribution, highlighting the utility of histogram-derived features in predicting molecular subtypes. This suggests that the nuclear DNA distribution data obtained from iFC provide valuable information beyond that captured by imaging alone.

Beyond Random Forest, we also examined feature importance using TabNet (Supplementary Figure 1). Notably, the top-ranked features—histogram6, TNR, patient age, T2-FLAIR mismatch, and gadolinium enhancement—were largely consistent between both classifiers, indicating a stable feature selection process. This agreement reinforces the reliability of these features across different interpretable models.

**Figure 4:**
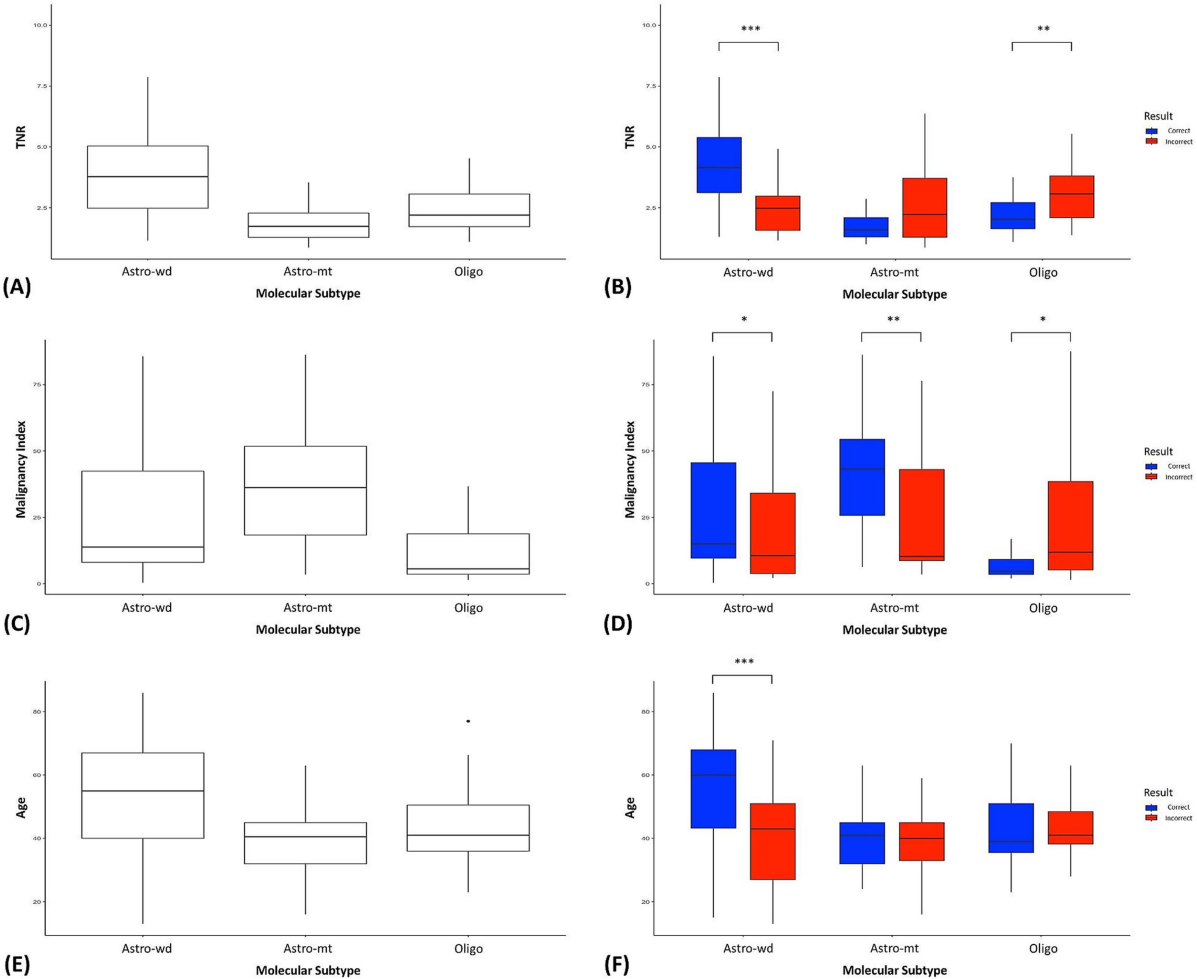
Comparison of key predictive variables across molecular subtypes and classification performance. **(A)** Distribution of tumor-to-normal uptake ratio (TNR) from ^11^C-methionine positron emission tomography across the three molecular subtypes (Astro-WD, Astro-MT, and Oligo). **(B)** TNR values stratified by classification outcome (correct = blue, incorrect = red) within each molecular subtype. Significant differences were observed for Astro-WD (^***^p < 0.001) and Oligo (^**^p < 0.01). **(C)** Distribution of malignancy index (MI) derived from intraoperative flow cytometry for each subtype. **(D)** Subtype-specific MI values categorized by prediction accuracy. Significant differences were found in Astro-WD (^*^p < 0.05), Astro-MT (^**^p < 0.01), and Oligo (^*^p < 0.05). **(E)** Patient age distribution across subtypes. **(F)** Age stratified by correct and incorrect model predictions within each subtype. Astro-WD showed significant differences (^***^p < 0.001). Box plots indicate median, interquartile ranges, and outliers. Significance levels: ^*^p < 0.05, ^**^p < 0.01, and ^***^p < 0.001.

Furthermore, a comparison between correctly classified and misclassified cases revealed significant differences in TNR, MI, and age for specific subtypes (Figures 4A–F and Table 3). For example, TNR and age differed significantly in Astro-WD, whereas MI was significantly different in both Astro-MT and Oligo. These results indicate that the model relies on clinically meaningful variables to guide classification.

**Table 3:** Comparison of key predictive features between correctly and incorrectly classified cases by molecular subtype.

Variable	Subtype	*n* (correct)	*n* (incorrect)	Median (correct)	Median (incorrect)	*U*-value	*p*-value
TNR	Astro-WD	59	23	4.15	2.48	1,039	0.0002
	Astro-MT	44	14	1.6	2.23	240	0.22
	Oligo	54	19	2.03	3.07	273	0.00255
MI	Astro-WD	114	27	15	10.6	1,986	0.0194
	Astro-MT	49	19	43.3	10.3	676	0.00419
	Oligo	55	24	4.67	11.9	437	0.0177
Age	Astro-WD	114	27	60	43	2,358	1.8 × 10^−5^
	Astro-MT	49	19	41	40	469	0.967
	Oligo	55	24	39	41	580	0.396

The table summarizes the differences in the tumor-to-normal uptake ratio (TNR), malignancy index (MI), and patient age between correctly predicted cases (OK) and misclassified cases (NG) for each molecular subtype (Astro-WD, Astro-MT, and Oligo). Statistical significance was assessed using Wilcoxon rank-sum test. Significant differences were observed for TNR and age in the Astro-WD group and for MI in the Astro-MT and Oligo groups. A-WD, IDH-wild-type astrocytoma; A-MT, IDH-mutant astrocytoma; and Oligo, oligodendroglioma.

Among the three molecular subtypes, oligodendroglioma demonstrated the lowest *F*_1_-score (0.68), with 22 out of 77 cases misclassified—primarily as Astro-WD, and to a lesser extent as Astro-MT (Table 2). Misclassified Oligo cases exhibited significantly higher MET-PET uptake (median TNR = 3.07 vs. 2.03, *p* = 0.0026) and malignancy index (11.9 vs. 4.67, *p* = 0.018) compared to correctly classified cases, while patient age did not differ significantly (*p* = 0.396) (Table 3). These findings suggest that some Oligo tumors may share biological features with Astro-WD, such as increased metabolic activity or abnormal nuclear DNA content, making classification more challenging. Further refinement of the model or inclusion of additional molecular markers may be required to improve prediction accuracy in such cases.

### 3.3 Development of intraoperative prediction application

We developed a prototype real-time prediction application based on the Random Forest model for intraoperative support. The application used preoperative imaging and intraoperative iFC data as inputs and outputs molecular subtype predictions in the form of probability distributions, which were visualized as pie charts for intuitive interpretation.

This tool enabled real-time prediction of the genetic subtype during surgery and supported intraoperative decision-making, such as determining the EOR. The user interface was designed to be intuitive and informative for both surgeons and support staff, allowing quick comprehension of the predictive results.

The model inference was completed in less than one second on a Microsoft Surface Go 2 (Intel Core m3-8100Y, 8GB RAM), resulting in negligible latency. In the current workflow, radiological features are assessed and entered preoperatively, and intraoperative flow cytometry results are available approximately 10 min after tissue sampling. Data transfer is performed via SD card, although direct network integration is under consideration. Taking into account data entry and transfer time, the entire process from sampling to prediction output typically takes about 15 min, which is well within the intraoperative decision-making window.

### 3.4 Illustrative cases

#### 3.4.1 Illustrative case 1

**Figure 5:**
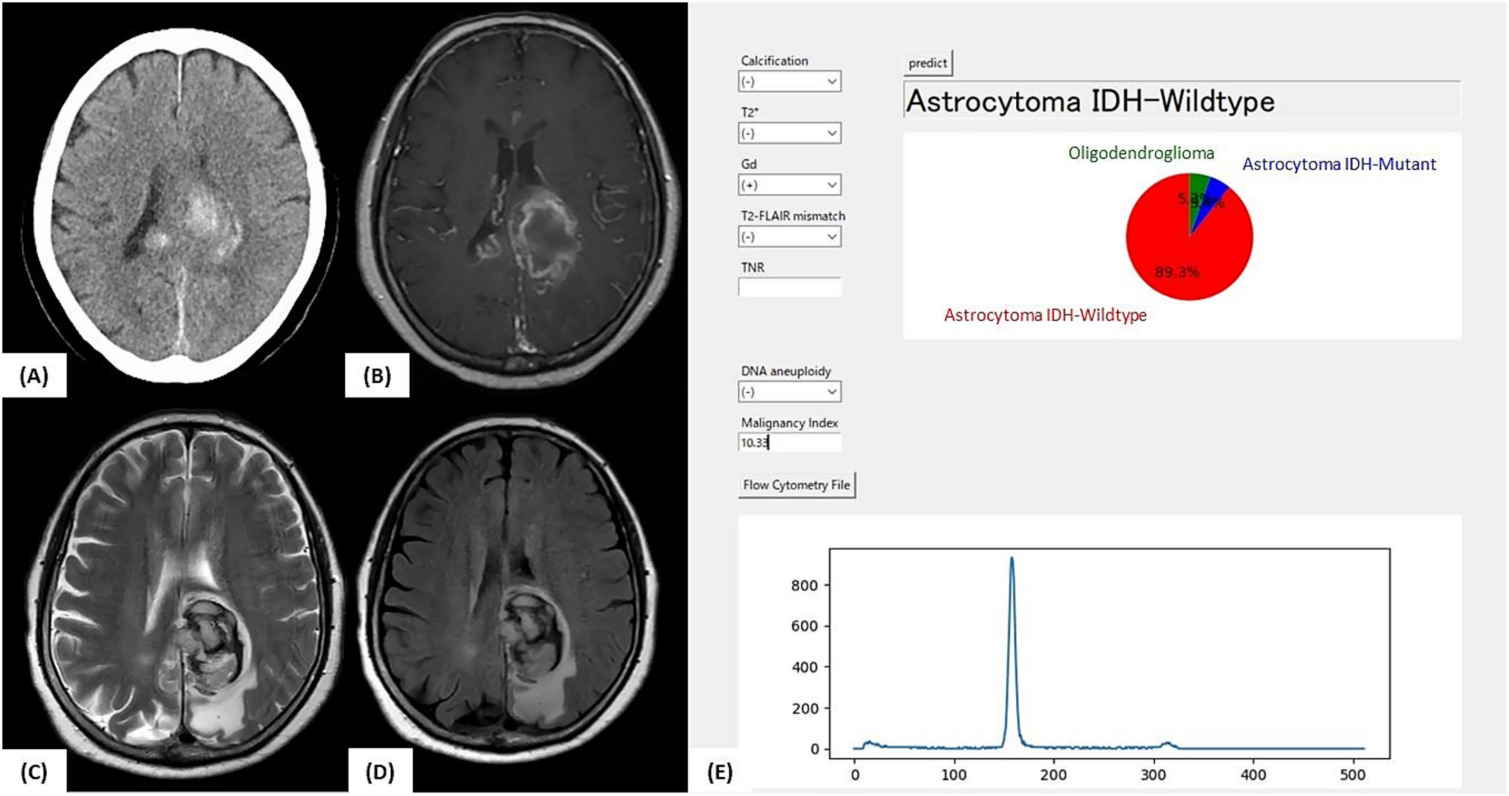
Illustrative case 1: Intraoperative prediction consistent with final diagnosis. **(A)** Preoperative CT image showing intratumoral hemorrhage without clear evidence of calcification. **(B)** Gadolinium-enhanced T1-weighted MRI showing contrast-enhancing lesions in the left parietal lobe and splenium of the corpus callosum. **(C)** T2-weighted MRI and **(D)** FLAIR image showing high-signal lesions without a T2-FLAIR mismatch. **(E)** Intraoperative prediction result generated by the developed support tool. Based on preoperative imaging and intraoperative flow cytometry findings (aneuploidy-negative, malignancy index = 10.3), the application predicted “Astrocytoma, IDH-wild-type” with a probability of 89.3%. The final molecular diagnosis was glioblastoma, IDH-wild-type, WHO CNS grade 4, consistent with the intraoperative prediction.

A 76-year-old woman presented with right-sided hemiparesis. MRI revealed contrast-enhancing lesions in the left parietal lobe and right splenium and FLAIR hyperintensity in the left temporal lobe (Figure 5). No T2-FLAIR mismatch or calcifications were observed. Intratumoral hemorrhage was present, suggesting a high-grade glioma.

The patient underwent tumor resection under general anesthesia. When preoperative imaging and intraoperative iFC data were input into the prediction application, the model predicted “Astro-WD” with the highest probability. Postoperative molecular diagnosis confirmed IDH wild-type, absence of 1p/19q co-deletion, and presence of a pTERT mutation, consistent with GBM, IDH wild-type, and WHO CNS grade 4. This case exemplifies a scenario in which the intraoperative prediction matches the final diagnosis, underscoring the tool’s clinical utility.

#### 3.4.2 Illustrative case 2

**Figure 6:**
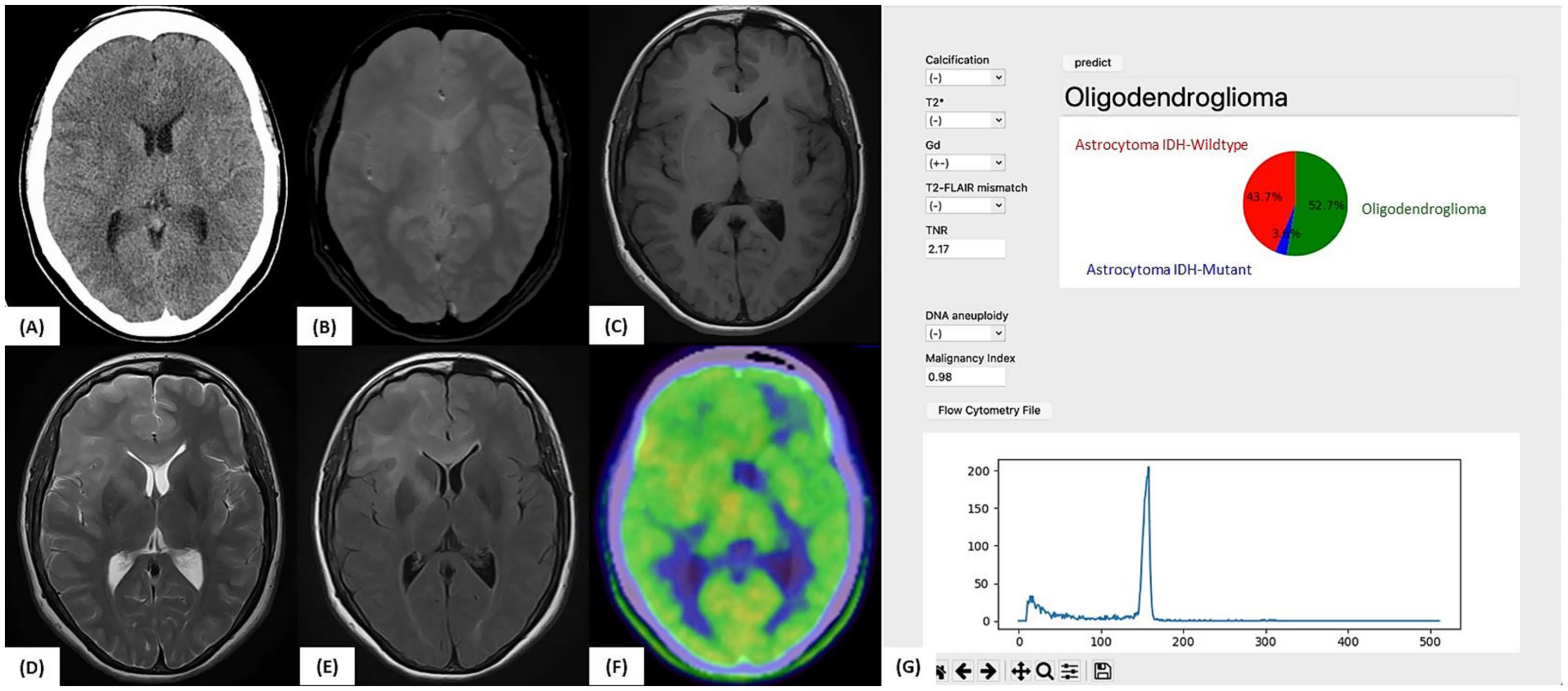
Illustrative case 2: Artificial intelligence prediction exceeding preoperative expectations. **(A)** Preoperative CT and **(B)** T2*-weighted MRI showing no evidence of calcification. **(C)** Gadolinium-enhanced T1-weighted MRI without any contrast enhancement. **(D)** T2-weighted MRI and **(E)** FLAIR image showing diffuse high-signal lesions without a T2-FLAIR mismatch. **(F)** ^11^C-methionine PET showing mildly increased tracer uptake (tumor-to-normal uptake ratio = 2.17). **(G)** Intraoperative prediction result generated by the support tool. Based on preoperative imaging and intraoperative flow cytometry data (aneuploidy-negative, malignancy index = 0.98), the predicted molecular subtype was “Oligodendroglioma” (52.7%), followed by “Astrocytoma, IDH-wild-type” (43.7%). The final molecular diagnosis confirmed oligodendroglioma, WHO CNS grade 2, with an IDH1 mutation, 1p/19q co-deletion, and TERT promoter mutation, consistent with the intraoperative AI-based prediction.

A 32-year-old woman underwent MRI for headache, which revealed a diffuse FLAIR hyperintense lesion extending from the right frontal lobe to the left frontal lobe via the corpus callosum, raising suspicion of gliomatosis cerebri (Figure 6). No apparent calcification, T2-FLAIR mismatch, or contrast enhancement was observed. Initial Met-PET showed mild uptake (TNR = 1.54).

After pregnancy, the lesion enlarged, prompting a stereotactic biopsy of the right frontal lobe and corpus callosum. A second Met-PET scan showed elevated uptake (TNR = 2.17) in the subcortical region of the right frontal lobe. Although Astro-WD was suspected based on imaging, iFC analysis revealed no aneuploidy and a low MI (0.98). The application predicted “Oligo: 52.7%, Astro-WD: 43.7%.”

Postoperative genetic testing confirmed the IDH1 R132H mutation, 1p/19q co-deletion, and pTERT mutation, resulting in a final diagnosis of oligodendroglioma, WHO CNS grade 2. This case demonstrates how artificial intelligence (AI)-assisted prediction provides accurate classification, even when clinical impressions differ, highlighting its value as an intraoperative support tool.

## 4 Discussion

In this study, we developed a machine learning model that integrates preoperative imaging data (MRI, CT, and ^11^C-MET PET) with iFC features to enable the real-time intraoperative prediction of molecular subtypes of glioma. The model achieved a high overall accuracy of 76.0% and ROC-AUCs of 0.88 (macro) and 0.89 (micro), indicating its potential as a practical intraoperative decision-support tool.

### 4.1 Novelty and clinical relevance

The novelty of our model lies in the integration of quantitative features derived from iFC, such as the MI, aneuploidy, and DNA histograms, with conventional imaging data for molecular classification. Although iFC has traditionally been used for rapid intraoperative assessments of malignancy and tumor typing, our approach demonstrates that these data can be quantitatively incorporated into machine learning algorithms to enhance diagnostic precision beyond that provided by imaging alone (15–17, 24, 25).

Our previous study proposed an intraoperative surgical strategy based on the molecular data obtained during surgery (18). This study expands on this concept by providing an AI-assisted framework for real-time predictions. Recent advances in intraoperative genetic testing, including real-time and digital droplet polymerase chain reaction, have highlighted the clinical utility of intraoperative molecular diagnostics (26, 27).

### 4.2 Feature interpretability and predictive factors

Feature importance analysis based on Gini impurities revealed that the TNR on ^11^C-MET PET, MI, patient age, bin 6 from DNA histograms, and gadolinium enhancement were the most influential predictors (Figure 3). The TNR, a known indicator of tumor metabolic activity, is associated with tumor grade and malignancy (28). MI reflects abnormalities in DNA content and has been linked to tumor aggressiveness and prognosis (15–18). Age has also been reported as a prognostic factor, particularly in lower-grade astrocytomas (28), and was notably lower in misclassified Astro-WD cases in our analysis.

The substantial contribution of histogram-derived features—especially bin 6—suggests that nuclear DNA distribution profiles captured by iFC contain valuable information that is not readily apparent from imaging alone.

In addition, comparisons between correctly and incorrectly classified cases showed statistically significant differences in the T/N ratio, MI, and age for certain subtypes (Figures 4A–F and Table 3). For instance, Astro-WD misclassification was associated with lower TNR and younger age, whereas Astro-MT and Oligo misclassifications were related to MI values, indicating that these variables were central to the model’s decision-making process.

This agreement reinforces the reliability of these features across different interpretable models.

To contextualize our model choice, we additionally evaluated LightGBM and XGBoost, which achieved accuracies of 0.72 and 0.73, respectively. These values were comparable to our primary models, while a baseline SVM classifier showed lower accuracy (0.64). In summary, Random Forest demonstrated the highest accuracy in our study. This finding implies that Random Forest may offer enhanced robustness to overfitting relative to gradient boosting and deep learning approaches, as well as improved adaptability to multiclass and imbalanced classification tasks compared to SVM.

### 4.3 Subtype-specific performance and challenges

Among the three molecular subtypes, the model performed least accurately for Oligo, with an *F*_1_-score of 0.68. This may be partly due to the overlap in imaging characteristics, such as elevated ^11^C-MET PET uptake, with Astro-WD. The iFC-derived features also showed limited specificity in a subset of Oligo cases.

Nevertheless, in illustrative case 2, the model accurately predicted an oligodendroglioma despite the initial suspicion as IDH wild-type based on imaging and clinical impression. This highlights the utility of iFC data as a complementary input when imaging-based assessments are inconclusive.

### 4.4 Clinical implications and broader significance

This study extended our previous research on iFC for intraoperative grading and tumor classification by demonstrating its applicability to molecular subtyping. The development of a functional application based on our model represents a meaningful step toward real-time intraoperative implementation.

Future integration with deep learning models and radiomics features derived from preoperative imaging could further enhance the diagnostic performance (29, 30). Our model may serve as a benchmark for the next generation of intraoperative decision-support systems for glioma surgery.

### 4.5 Limitations and future directions

This retrospective study was conducted at a single institution. Thus, external validation and further case accumulation are essential to ensure generalizability. Some misclassified cases show atypical imaging or iFC patterns, underscoring the potential need for new markers or additional molecular data in select cases.

In the future, the integration of real-time quantitative data, including iFC and intraoperative genetic diagnostics, may enable the prediction of not only molecular subtypes, but also tumor grading, further supporting precise surgical decision-making.

Finally, we note that this study focused solely on molecular subtype classification and did not evaluate glioma grading (i.e., low vs. high grade) using ROC analysis. Although the malignancy index has been associated with tumor grade in prior work (16), further studies will be needed to extend this model to real-time grading support.

## 5 Conclusion

In this study, we developed a machine learning model that integrates preoperative imaging data with iFC features to enable the real-time prediction of molecular subtypes of glioma. The model demonstrated high predictive accuracy and clinical relevance, with the TNR on ^11^C-MET PET, MI, and patient age identified as key predictive factors. These findings highlight the contribution of quantitative iFC data to improving diagnostic precision beyond imaging alone.

Furthermore, the development of a user-friendly intraoperative prediction application based on this model indicates its feasibility for clinical implementation. With continued case accumulation, external validation, and future integration with deep-learning approaches, this framework may eventually support the intraoperative prediction of molecular subtypes and tumor grading. Such advancements would directly contribute to optimizing surgical strategies, including the EOR, and promote personalized treatment planning during glioma surgery.

## Data Availability

The datasets generated or analyzed during the current study contain sensitive clinical imaging and intraoperative data and are therefore not publicly available due to ethical and privacy considerations. However, de-identified data may be made available from the first author, Dr. Shunichi Koriyama, upon reasonable request and with prior approval from the institutional ethics committee.
