# Supplementary material for: High-precision intraoperative diagnosis of gliomas: integrating imaging and intraoperative flow cytometry with machine learning
Source: Front Neurol. 2025 Sep 9;16:1647009. doi: 10.3389/fneur.2025.1647009 (PMC12454067; doi:10.3389/fneur.2025.1647009)
Supplement: Supplementary file 1 [file Data_Sheet_1.PDF]

Supplementary Table 1. Confusion Matrices of Restricted Models

A. Imaging + Age model		predict		
		A-WD	A-MT	Oligo
Molecular Subtype	A-WD	101	10	30
	A-MT	10	41	17
	Oligo	14	16	49

B. iFC + Age model		predict		
		A-WD	A-MT	Oligo
Molecular Subtype	A-WD	109	11	21
	A-MT	12	46	10
	Oligo	16	10	53

C. Imaging-only model		predict		
		A-WD	A-MT	Oligo
Molecular Subtype	A-WD	103	9	29
	A-MT	9	46	13
	Oligo	14	16	49

Confusion matrices for restricted models used to evaluate modality contributions. A: imaging + age (overall accuracy = 66 %) B: iFC + age (overall accuracy = 72 %) C: imaging-only (overall accuracy = 69 %). A-WD, IDH-mutant astrocytoma; A-MT, IDH mutant astrocytoma; and Oligo, oligodendroglioma

Supplementary Figure 1.

Feature importance ranking in the TabNet model based on aggregated attention weights. The most influential features were histogram6, patient age, TNR, and T2–FLAIR mismatch, followed by gadolinium enhancement. These top-ranked features were largely consistent with those identified by the Random Forest model, suggesting a stable feature selection process across different interpretable classifiers.
