# Supplementary figures and images for: High-precision intraoperative diagnosis of gliomas: integrating imaging and intraoperative flow cytometry with machine learning
Source: Front Neurol. 2025 Sep 9;16:1647009. doi: 10.3389/fneur.2025.1647009 (PMC12454067; doi:10.3389/fneur.2025.1647009)

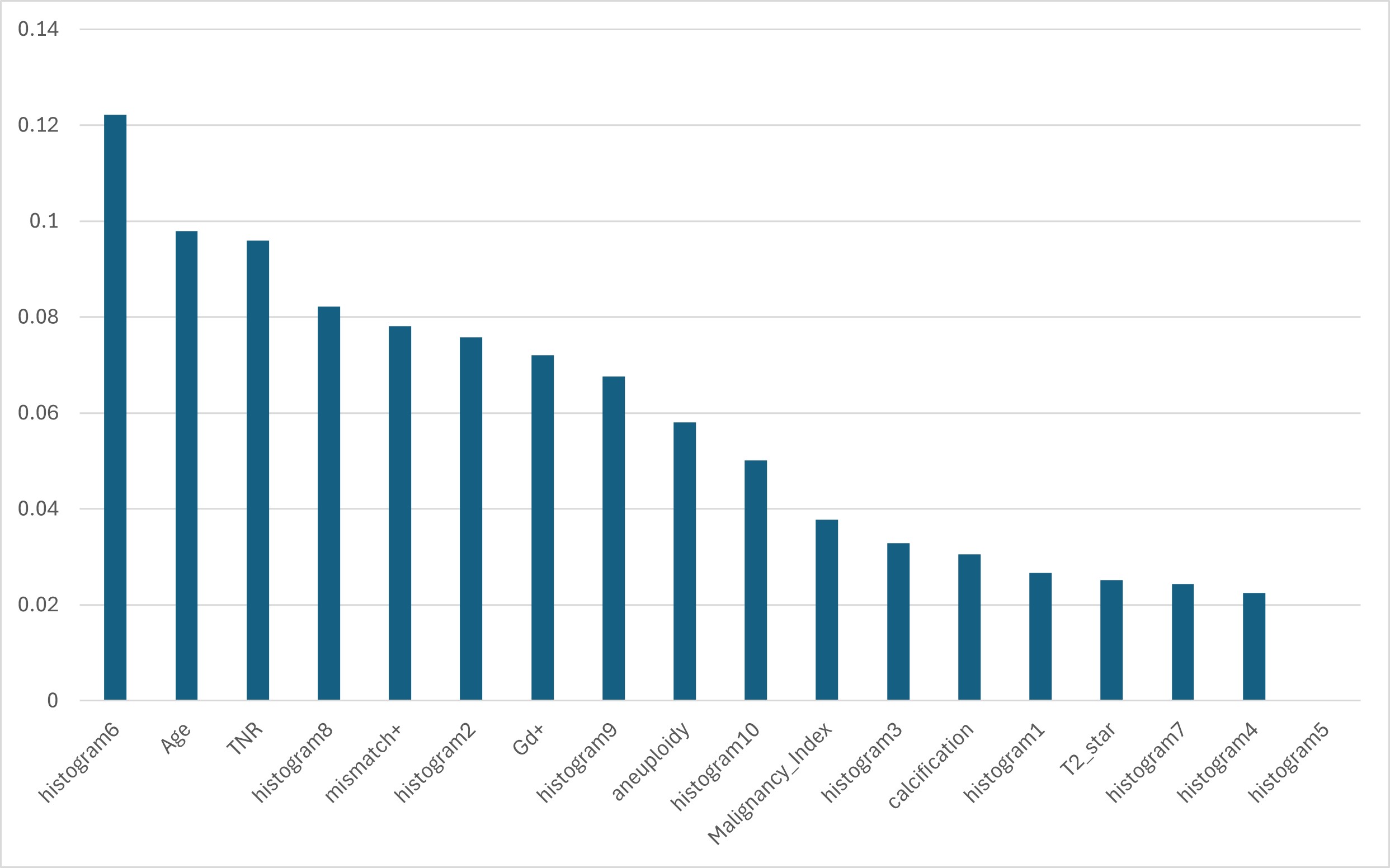

Supplement: Supplementary file 2 [file Image_1.JPEG]
